# Suppressing activity of tributyrin on hepatocarcinogenesis is associated with inhibiting the p53-CRM1 interaction and changing the cellular compartmentalization of p53 protein

**DOI:** 10.18632/oncotarget.8248

**Published:** 2016-03-22

**Authors:** Juliana F. Ortega, Aline de Conti, Volodymyr Tryndyak, Kelly S. Furtado, Renato Heidor, Maria Aderuza Horst, Laura Helena Gasparini Fernandes, Paulo Eduardo Latorre Martins Tavares, Marta Pogribna, Svitlana Shpyleva, Frederick A. Beland, Igor P. Pogribny, Fernando Salvador Moreno

**Affiliations:** ^1^Laboratory of Diet, Nutrition and Cancer, Department of Food and Experimental Nutrition, Faculty of Pharmaceutical Sciences, University of São Paulo, São Paulo, Brazil; ^2^Division of Biochemical Toxicology, National Center for Toxicological Research, Jefferson, USA

**Keywords:** hepatocarcinogenesis, chemoprevention, tributyrin, p53 subcellular localization, CRM1

## Abstract

Hepatocellular carcinoma (HCC), an aggressive and the fastest growing life-threatening cancer worldwide, is often diagnosed at intermediate or advanced stages of the disease, which substantially limits therapeutic approaches for its successful treatment. This indicates that the prevention of hepatocarcinogenesis is probably the most promising approach to reduce both the HCC incidence and cancer-related mortality. In previous studies, we demonstrated a potent chemopreventive effect of tributyrin, a butyric acid prodrug, on experimental hepatocarcinogenesis. The cancer-inhibitory effect of tributyrin was linked to the suppression of sustained cell proliferation and induction of apoptotic cell death driven by an activation of the p53 apoptotic signaling pathway. The goal of the present study was to investigate the underlying molecular mechanisms linked to tributyrin-mediated p53 activation. Using *in vivo* and *in vitro* models of liver cancer, we demonstrate that an increase in the level of p53 protein in nuclei, a decrease in the level of cytoplasmic p53, and, consequently, an increase in the ratio of nuclear/cytoplasmic p53 in rat preneoplastic livers and in rat and human HCC cell lines caused by tributyrin or sodium butyrate treatments was associated with a marked increase in the level of nuclear chromosome region maintenance 1 (CRM1) protein. Mechanistically, the increase in the level of nuclear p53 protein was associated with a substantially reduced binding interaction between CRM1 and p53. The results demonstrate that the cancer-inhibitory activity of sodium butyrate and its derivatives on liver carcinogenesis may be attributed to retention of p53 and CRM1 proteins in the nucleus, an event that may trigger activation of p53-mediated apoptotic cell death in neoplastic cells.

## INTRODUCTION

Hepatocellular carcinoma (HCC), an aggressive and life-threatening cancer in humans, is the fastest growing cause of cancer-related deaths in the United States [1] and the third most common cause of death from cancer in the world [2]. HCC develops in a milieu of interconnected molecular alterations in fundamental signaling pathways, among which a disruption of the balance between cell proliferation and cell death driven by enhanced cell replication and the suppression of apoptosis is the central cancer-related principle of both human and rodent liver carcinogenesis [3, 4]. This suggests that strategies focusing on the inhibition of anti-apoptotic signaling and/or activation of apoptotic cell death may have the potential to improve the clinical management of HCC.

Butyric acid, a product of the fermentation of dietary fibers by endogenous bacteria, is considered a promising anti-cancer agent [5]; however, its limited bioavailability, short half-life, and difficulty in producing effective plasma concentrations compromise its therapeutic application. In contrast, tributyrin, a butyric acid prodrug, present in milk fat and honey [6], exhibits a more favorable plasma half-life and greater anti-cancer potential in *in vitro* and *in vivo* preclinical studies [7–9].

In previous studies using a classic “resistant hepatocyte” model of rat liver carcinogenesis [10] that recapitulates the development of human HCC [11], we demonstrated that treatment of rats undergoing hepatocarcinogenesis with tributyrin during the initiation and promotion stages inhibited the carcinogenic process via the induction of apoptotic cell death in preneoplastic enzyme-altered foci [12, 13]. Furthermore, Watkins *et al.* [14] reported that tributyrin treatment induces apoptosis in human liver cancer cells, and Yamamoto *et al.* [15] and Emanuele *et al.* [16] demonstrated increased apoptotic cell death in human liver cancer cells treated with sodium butyrate. The anti-cancer activity of tributyrin was attributed to its activity to act as a histone deacetylase inhibitor and inducer of the expression of pro-apoptotic genes in the p53 signaling pathway [13].

The tumor suppressor protein, p53, is a fundamental regulator of apoptosis in response to exogenous and endogenous stimuli [17]. The presence of p53 in the nucleus of the cells is essential to its functional activity and this is under control of nuclear-cytoplasmic translocation mechanisms [18], including factors related to p53 nuclear import and export, subnuclear localization, fixation, and cytoplasmic sequestration [19, 20]. Aberrant cytoplasmic accumulation of p53 has been linked to alterations in cellular differentiation, increased malignancy, tumor progression, metastases, cancer drug resistance, and poor prognosis [21]. Previously, we [12, 13] demonstrated that treatment with tributyrin during the initiation and promotion stages of liver carcinogenesis reduced cytoplasmic accumulation of p53 in preneoplastic livers indicating the possibility that tributyrin influences the subcellular localization of p53; however, the underlying mechanism of this phenomenon remained unexplored.

Based on these considerations, in the present study we investigated (*i*) the chemopreventive effect of tributyrin when administered only during the promotion stage of hepatocarcinogenesis, and (*ii*) the potential mechanism of tributyrin-induced retention of p53 protein in nuclei by using *in vitro* and *in vivo* models of liver carcinogenesis. We demonstrate that treatment of rats with tributyrin during the promotion stage of liver carcinogenesis results in a large increase in apoptotic cell death in glutathione-*S*-transferase placental form (GST-P)-positive preneoplastic foci, as well as in rat HCC JM1 cells treated with sodium butyrate. These changes were accompanied by a substantial increase in the nuclear level of p53 protein in the preneoplastic livers of rats treated with tributyrin and in rat and human HCC cell lines treated with sodium butyrate. We demonstrate that elevation of p53 protein in the nuclei is paradoxically associated with an increase in the nuclear level of chromosome region maintenance 1 (CRM1; Exportin1; XPO1) protein, the main nuclear translocation protein involved in exporting p53 protein to the cytoplasm [22], in preneoplastic livers and rat and human HCC cell lines treated with either tributyrin or sodium butyrate. Mechanistically, this effect may be attributed to an ability of tributyrin and sodium butyrate to reduce markedly the binding interaction of CRM1 with p53 and, thus, favor the accumulation of p53 protein in the nucleus and its reduction in the cytoplasm.

## RESULTS

### Effect of tributyrin on the formation of preneoplastic lesions

Immunohistochemical analysis of liver sections of rats undergoing liver carcinogenesis revealed the presence of large preneoplastic GST-P-positive foci that were evenly distributed throughout the entire section of the liver. In the livers of rats treated with tributyrin during the promotion stage of carcinogenesis, the size of the GST-P positive foci, as well as the area of liver occupied by them, were significantly smaller (*P* < 0.05) than in control rats (Table 1).

**Table 1:** Morphometric analysis of GST-P positive foci in the livers of rats subjected to a“resistant hepatocyte” model of hepatocarcinogenesis (control group) and treated with tributyrin during the promotion phase

Groups (*n*)	Number of GST-P positive PNL per cm^2^	Size of GST-P positive PNL (mm^2^)	Area of liver section occupied by GST-P positive PNL (%)	BrdU positive hepatocytes/mm^2^ PNL	Hepatic apoptotic bodies/mm^2^ PNL
Control (6)	61 ± 4	0.38 ± 0,09	25 ± 2	12.11 ± 5.85	0.37 ± 0.34
Tributyrin (8)	55 ± 4	0.19 ± 0.01*	10 ± 1*	6.68 ± 6.64	2.23 ± 0.89*

* Statistically significant difference (*P* < 0.05) when compared to the control group. Values are means ± S.D. PNL - preneoplastic lesion.

### Effect of tributyrin on the extent of cell proliferation and apoptosis

A double-labeling immunohistochemical staining approach was used to examine the extent of cell proliferation in the preneoplastic GST-P-positive foci in the livers of control and rats treated with tributyrin. Table 1 shows that treatment of rats undergoing hepatocarcinogenesis with tributyrin did not affect the extent of cell proliferation, as measured by BrdU positive hepatocytes. In contrast, the extent of apoptosis in the GST-P-positive foci in tributyrin-treated rats was six times greater than in untreated control rats (Table 1).

**Figure 1:**
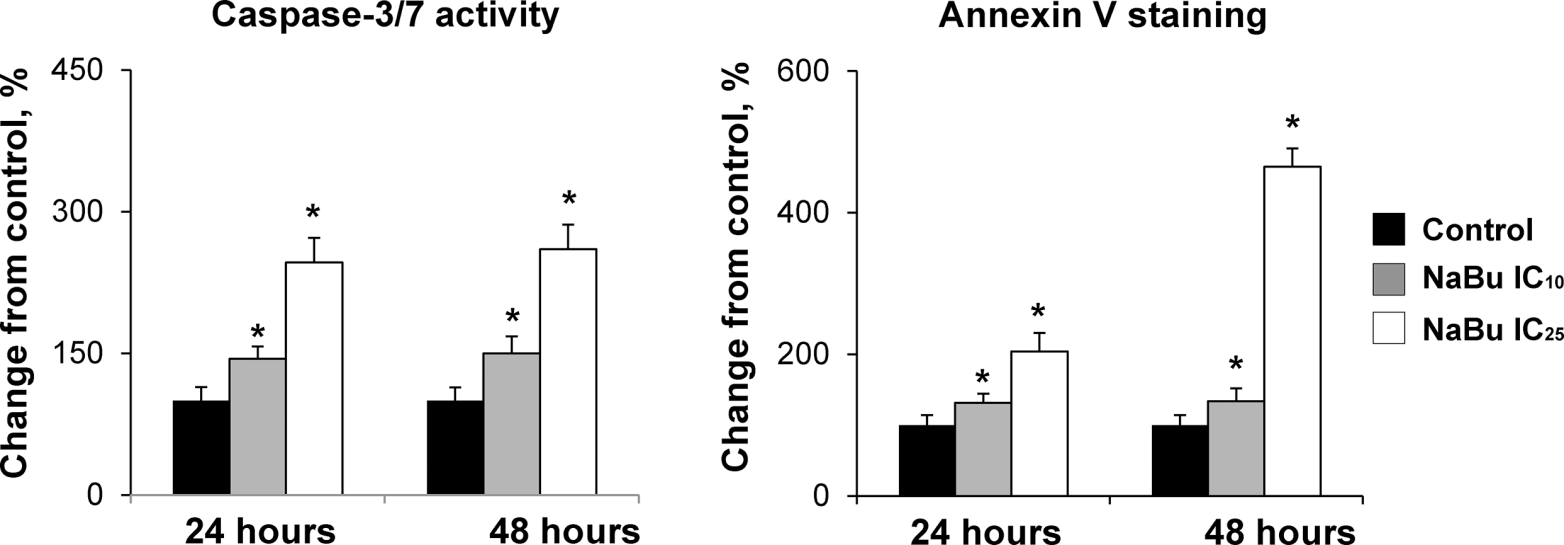
Apoptosis in the rat HCC JM1 cells The extend of apoptotic cell death in rat HCC JM1 control cells and cells treated with sodium butyrate was analyzed by annexin-V/propidium iodide staining and caspases 3/7 activity assays. The experiments were conducted in triplicate. * - Significantly different from control group.

To investigate further the effect of tributyrin on cell proliferation and apoptosis, rat HCC JM1 cells, a cell line derived from a primary HCC induced in a partially hepatectomized Fischer 344 rat that had received a single dose of *N*-nitrosodiethylamine (DEN) followed by chronic phenobarbital administration [23], were treated with sodium butyrate. Similar to the *in vivo* data, treatment of the JM1 cells *in vitro* with sodium butyrate at IC_10_ and IC_25_ doses did not affect cell proliferation (data not shown), whereas the extent of apoptosis was greatly increased, evidenced by an increased caspase 3/7 activity and annexin V staining (Figure 1).

### Effect of tributyrin and sodium butyrate on the level and localization of p53 protein

**Figure 2:**
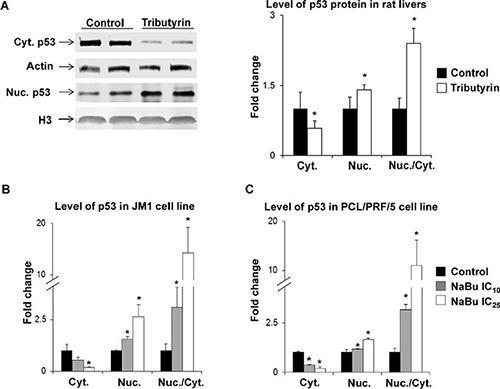
Subcellular localization of p53 protein in preneoplastic livers in rats undergoing hepatocarcinogenesis and in HCC cell lines Western blot analysis of p53 in cytoplasmic and nuclear fractions from liver tissue of control rats (*n* = 5) and rats treated with tributyrin (*n* = 5) (**A**) and in cytoplasmic and nuclear fractions in the rat HCC JM1 (**B**) and human HCC PLC/PRF/5 (**C**) control cells and cells treated with sodium butyrate (*n* = 3 per treatment group). The results are presented as an average fold change in the level of cytoplasmic or nuclear p53 protein in the livers of tributyrin-treated rats or sodium butyrate-treated cells relative to their respective controls, which were assigned a value 1. Cytoplasmic and nuclear levels of p53 were normalized to β-actin or histone H3, respectively. Values are means ± S.D. * - Significantly different from control group.

In our previous studies on rat liver carcinogenesis, we demonstrated that treatment of rats during the initiation and promotion stages of hepatocarcinogenesis with tributyrin resulted in activation of pro-apoptotic genes in the p53 signaling pathway driven by an increased accumulation of p53 protein in the nucleus [13] and a decreased level of this protein in the cytoplasm [12, 13]. Figure 2 shows that the treatment of rats undergoing liver carcinogenesis *in vivo* with tributyrin during the promotion stage resulted in a reduction in the cytoplasmic level of p53 protein and increase in the nuclear level of p53 protein (Panel A). This caused a substantial increase in ratio of nuclear to cytoplasmic fractions of p53 protein in the preneoplastic livers. Similar changes in the subcellular p53 localization were found in the rat HCC JM1 cells and human HCC PLC/PRF/5 cells treated with sodium butyrate (Figure 2, Panels B and C). PLC/PRF/5 cells harbor a AGG to AGT (arginine to serine) mutation at codon 249 (p.R249S) that accounts for 90% of TP53 mutations in aflatoxin B_1_-related HCC [24].

### Effect of tributyrin and sodium butyrate on the level and localization of CRM1 protein

**Figure 3:**
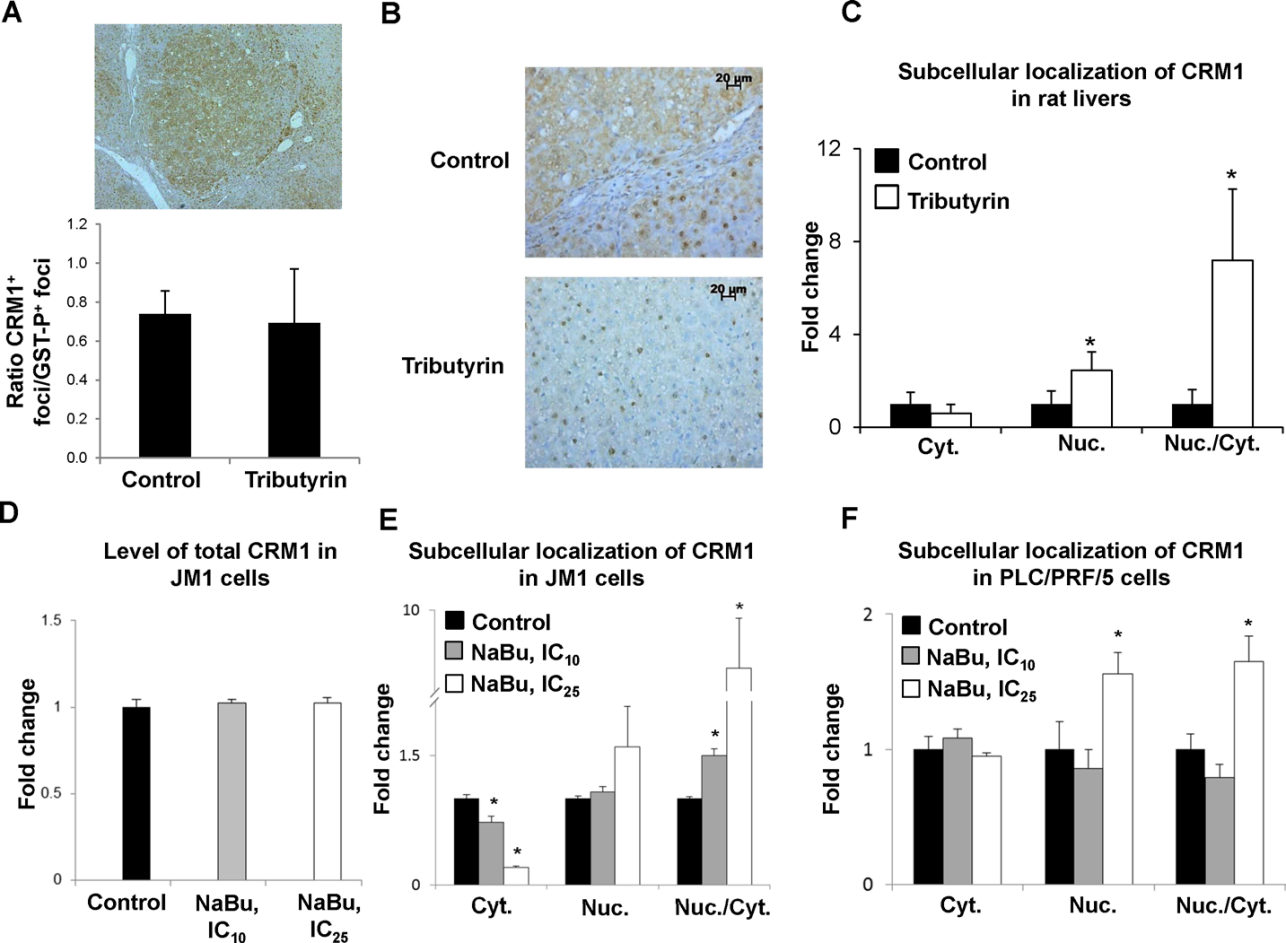
Subcellular localization of CRM1 protein in preneoplastic livers in rats undergoing hepatocarcinogenesis and in HCC cell lines Immunohistochemical analysis of CRM1 staining in GST-P-positive foci (**A**) Representative images of CRM1 cytoplasmic staining in liver tissues from control rats and CRM1 nuclear staining in rats treated with tributyrin (**B**) Fold change of GST-P-positive foci stained for cytoplasmic or nuclear CRM1 in the livers of control rats (*n* = 5) and rats treated with tributyrin (*n* = 5). (**C**) Western blot analysis of total CRM1 protein (**D**), and cytoplasmic and nuclear CRM1 protein levels in the rat HCC JM1 (**E**) and human HCC PLC/PRF/5 (**F**) control cells and cells treated with sodium butyrate (*n* = 3 per treatment group). Values are means ± S.D. * - Significantly different from control group.

In order to investigate the potential mechanism of tributyrin- and sodium butyrate-induced prevention of carcinogenesis associated with a decrease in p53 accumulation in the cytoplasm and a retention of p53 protein in the nuclei, the level of CRM1, the primary nuclear export protein involved in p53 protein translocation to cytoplasm [22, 25], was investigated. Immunohistochemical analysis of liver sections of rats revealed that the majority of GST-P-positive preneoplastic foci were positive for CRM1 (Figure 3, Panel A). The liver sections of rats undergoing liver carcinogenesis were characterized by a predominantly cytoplasmic CRM1 immunostaining (Figure 3, Panel B). Treatment of rats with tributyrin resulted in a 2-fold increase (*P* < 0.05) in the nuclear CRM1 protein staining in GST-p-positive preneoplastic foci (Figure 3, Panel C). Treatment of the rat HCC JM1 cells with sodium butyrate did not affect the total level of CRM1 protein (Figure 3, Panel D), but markedly reduced the level of CRM1 protein in the cytoplasm and increased its level in the nuclei in rat HCC JM1 and human HCC PLC/PRF/5 p53-mutant cells (Figure 3, Panels E and F). All of these subcellular CRM1 localization changes in the preneoplastic livers of both rat and human HCC lines resulted in a profound increase in ratio of nuclear to cytoplasmic forms of CRM1 protein (Figure 3, Panels C, E, and F).

### Inhibition of CRM1-p53 interaction by sodium butyrate

**Figure 4:**
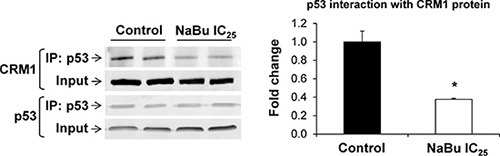
Interaction of p53 with CRM1 protein Co-immunoprecipitation and Western blot analyses were conducted in total cellular extracts from rat HCC JM1 control cells and cells treated with sodium butyrate. The experiments were conducted in triplicate. * - Significantly different from control group.

A large number of cancer-associated proteins, including p53, require CRM1 for their nuclear export [22]. In light of this, the interaction of CRM1 protein with p53 in the rat HCC JM1 cells, treated or untreated with sodim butyrate, was investigated. Figure 4 shows that treatment of rat HCC JM1 cells with sodium butyrate for 48 hr blocked the interaction between p53 and CRM1 by 50%.

### Expression of CRM1 in human HCC

**Figure 5:**
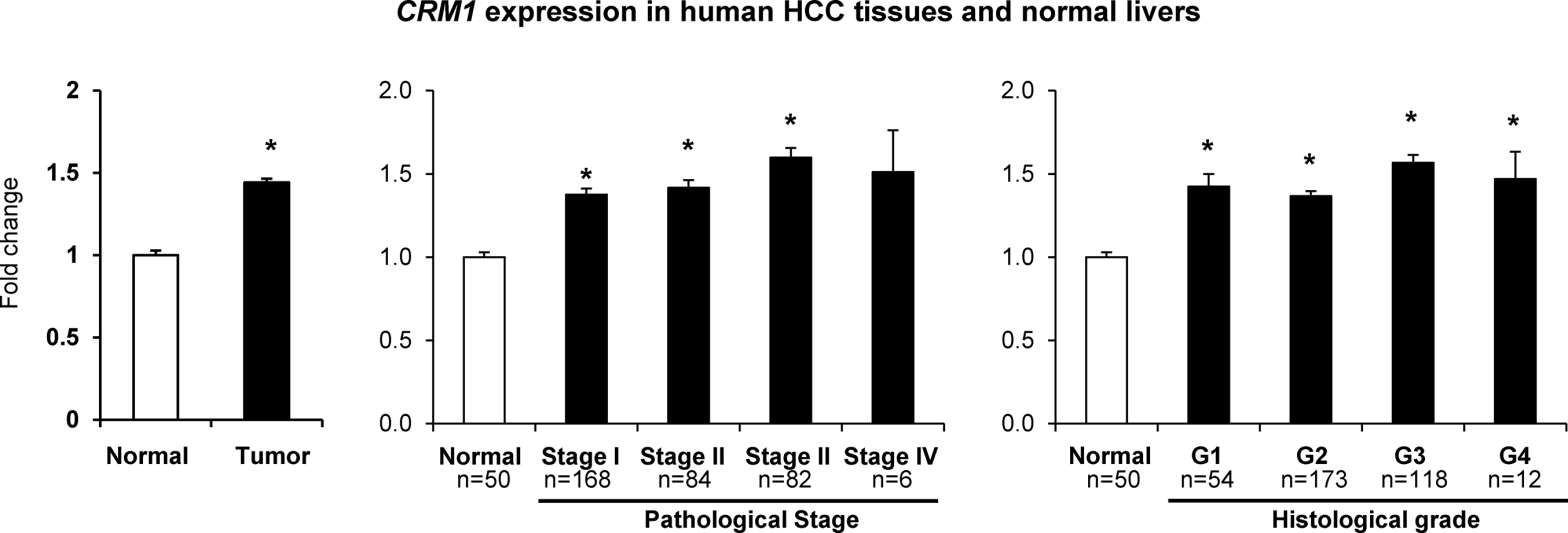
Expression of the *CRM1* gene in human HCC samples *CRM1* gene expression and clinical and tumor pathological data were extracted from the TCGA database.

The expression levels of the *CRM1* gene in human HCC tissue samples and normal liver samples were extracted from the TCGA database. Figure 5 shows that the expression of *CRM1* in HCC tissue samples was significantly greater than in normal livers.

## DISCUSSION

The results of several comprehensive studies have indicated that sodium butyrate and a butyric acid prodrug, tributyrin, exhibit a potent ability to prevent and/or inhibit carcinogenesis, including hepatocarcinogenesis. In previous studies [12, 13], we showed a potent inhibitory activity of tributyrin on rat hepatocarcinogenesis when administered during the initiation and promotion stages. In the present study, we demonstrate that the treatment of rats with tributyrin during the promotion stage of liver carcinogenesis greatly reduced the number, size, and area of the GST-P-positive preneoplastic foci. This inhibitory effect was associated with a marked activation of apoptotic cell death in GST-P-positive lesions and substantial elevation in the nuclear level of p53 protein in the preneoplastic livers.

p53 protein is an important regulator of cell proliferation, DNA damage repair, and apoptosis, the activities of which can be inhibited by p53's cellular compartmentalization [22, 26]. Specifically, an increased accumulation of p53 in the cytoplasm, where p53 protein is not functional, is related to an increased malignancy of several major human cancers, including HCC [26, 27]. Additionally, an elevation in cytoplasmic p53 protein, with a concomitant loss in nuclear staining, was observed in preneoplastic livers in early phases of hepatocarcinogenesis [26–29] and was linked to genomic instability and genetic alterations associated with malignant transformation [30]. In contrast, the cancer-inhibitory activity of several anti-cancer agents has been associated with concomitant induction of apoptosis and increased accumulation of p53 protein in the nuclei of the tumors [30–33]. Similar to those findings, the results of this study demonstrate that the increase in the nuclear level of p53 protein in preneoplastic livers of rats and in rat and human HCC cell lines treated with tributyrin or sodium butyrate was associated with markedly induced apoptotic cell death.

Several mechanisms may contribute to the dysregulation of an accurate maintenance of the proper intracellular compartmentalization of the p53 protein, among which an interaction of p53 with proteins that promotes its nuclear export, especially by nuclear exporter CRM1, may be one of the main mechanisms [22]. Over-expression of CRM1 has been found in many major human cancers and is related to tumor progression, increased tumor size, metastasis, cancer drug resistance, and a decrease in survival [34–36]. In the present study, we found an up-regulation and redistribution in intracellular compartmentalization of CRM1 in the GST-P-positive preneoplastic livers of rats undergoing liver carcinogenesis and over-expression of the *CRM1* gene in human primary HCC tissue samples. These findings are in a good agreement with a previous report that showed up-regulation of CRM1 in preneoplastic GST-P-positive foci, which correlated with progression of hepatocarcinogenesis in rats and in human HCC [37]. Therefore, it has been suggested that inhibition of the CRM1-mediated nuclear export and restoration of normal function of tumor suppressors, including p53, may be an effective universal approach for the treatment of cancer [25, 34]. Indeed, several drugs, known to be selective inhibitors of nuclear export, have been developed and tested in pre-clinical and human clinical cancer trial studies alone or in combination with other chemotherapeutic agents [25, 35, 36, 38].

A central finding of this study is the demonstration that the treatment of rats undergoing liver carcinogenesis with tributyrin during the promotion stage of the hepatocarcinogenic process, and rat and human HCC cells with sodium butyrate reduced the binding interaction between CRM1 and p53. This may lead to an increased nuclear accumulation of p53 protein and marked activation of p53-mediated apoptotic cell death in the preneoplastic foci and HCC cells. This finding is particularly important since the use of a number of CRM1 inhibitors in human clinical trials has been hammered by their high toxicity. In light of this, sodium butyrate and its derivatives, which are characterized by minimal side-toxic effects, may be an attractive approach for cancer intervention and cancer prevention, including HCC, in combination with current chemotherapeutic drugs. Nevertheless, future studies are needed to identify the molecular mechanisms associated with sodium butyrate- and tributyrin-mediated inhibition of p53-CRM1 interaction in cancer cells and their efficacy on the development and progression of HCC using different relevant model systems. Additionally, in the present study the chemopreventive activity of tributyrin on hepatocarcinogenesis was investigated in male rats because of the greater incidence of HCC in men than in women [2]. However, future research is required to investigate the sex-specific chemopreventive efficiency of tributyrin.

## MATERIALS AND METHODS

### *In vivo* rat model of hepatocarcinogenesis

Male Wistar rats (*n* = 14) were obtained from the colony of the Faculty of Pharmaceutical Sciences, University of São Paulo (São Paulo, Brazil), housed in a temperature-controlled room (24°C) with a 12 hr light-dark cycle, and given *ad libitum* access to water and commercial chow diet (Purina Nutrimentos Ltd, Paulinia, Brazil). After one week of adaptation, the animals were submitted to a “resistant hepatocyte model” of liver carcinogenesis, as previously described [10].

Briefly, the rats received a single intraperitoneal injection of DEN (Sigma-Aldrich, St Louis, MO; 200 mg/kg body weight; dissolved in 0.9% of NaCl) to initiate hepatocarcinogenesis. After a 2-week recovery period, the rats were gavaged with 2-acetylaminofluorene (2-AAF; Sigma-Aldrich; 20 mg/kg body weight; dissolved in corn oil) for four consecutive days, and then subjected to a partial hepatectomy [10]. One day after the partial hepatectomy, the rats were gavaged with 2-AAF (20 mg/kg body weight and allocated randomly to control and experimental groups. Rats (*n* = 8) in the experimental group were treated by gavage with tributyrin (2 g/kg body weight) daily for 5 consecutive weeks. Rats (*n* = 6) in the control group received maltodextrin (Nestlé, São Paulo, Brazil; 3 g/kg body weight) at an isocaloric dose to the tributyrin group. All animals were sacrificed 8 weeks after DEN administration. All experimental procedures were performed in accordance with an animal study protocol approved by the Faculty of Pharmaceutical Sciences of the University of São Paulo Ethics Committee for Animal Research (Protocol number 243).

### Cell lines and cell culture

The human HCC PLC/PRF/5 (p53 mutant p.R249S) cell line was obtained from the American Type Culture Collection (ATCC, Manassas, VA) and maintained in Williams' medium. The rat HCC JM1 cell line, derived from a primary hepatocellular carcinoma in a Fischer 344 rat [22], a gift by Dr. George Michalopoulos, University of Pittsburgh, (Pittsburgh, PA), was cultured in Dulbecco's modified Eagle's medium (Gibco, Carlsbad, CA) supplemented with 10% fetal bovine serum (FBS). Cells were seeded at a density 0.5 × 10^6^ viable cells per 100 mm plate, and the media was changed twice per week. The cells were scrapped onto ice, washed in phosphate-buffered saline (PBS), frozen at −80°C, or immediately utilized in subsequent analyses.

### Treatment of HCC cells with sodium butyrate

Sodium butyrate (Sigma-Aldrich, St. Louis, MO) was diluted in deionized water, and sterilized by filtration. JM1 and PLC/PRF/5 cells were treated with 0, 1, 2, 5, or 10 mM sodium butyrate. Control cells were treated with the same volume of sterile deionized water. After 48 hr of incubation, cell viability was analyzed by a trypan blue exclusion test [39]. The IC_10_ (inhibitory concentration to produce 10% cell death) and IC_25_, values were determined by using the resulting dose-response curves.

### Immunohistochemistry and image analysis

The status of GST-P, a well-accepted end-point indicator of rat liver carcinogenesis [40], was determined by immunohistochemistry, as described previously [12]. A dual-labeling immunohistochemistry technique was used to investigate the extent of cell proliferation in the GST-P-positive foci in livers of control and experimental rats as described in Mazzantini *et al.* [29]. Positive staining for CRM1 (1:200) protein was analyzed in serial histologic sections stained for GST-P.

### Apoptosis evaluation

The extent of apoptosis in the livers of control and tributyrin-treated rats was analyzed for the presence of apoptotic bodies as described previously [12]. Apoptosis in the rat HCC JM1 cells was evaluated by annexin-V/propidium iodide staining and caspases 3/7 activity assays. Cells were stained using an ApopNexin Annexin V FITC Apoptosis detection kit (EMD Millipore, Billerica, MA), according to the manufacturer's protocol and analyzed using a FACSort flow cytometer (Becton Dickinson, Washington, DC). Heat-shock treated cells were used as a positive control, and non-stained cells as a negative control. Quantitative analyses of caspases-3/7 activity in cells were conducted by ELISA, using Caspase-Glo^®^3/7 reagent (Promega, Madison, USA) as substrate, according to manufacturer's instructions.

### Western blot analysis of protein levels

Cytoplasmic and nuclear fractions from liver tissues samples (*n* = 5 per group) were prepared as described previously [13]. Cytoplasmic and nuclear fractions from HCC cells were prepared by using a Subcellular Protein Fractionation Kit for Cultured Cells (ThermoFisher Scientific, Grand Island, NY), according to the manufacturer's protocol. Extracts containing equal quantities of proteins were separated by sodium dodecyl sulfate-polyacrylamide gel electrophoresis on 8–15% polyacrylamide gels and transferred to polyvinylidene difluoride membranes. Membranes were probed with primary antibodies against p53 (1:500; Cell Signaling Technology, Danvers, MA), CRM1 (1:1000; Abcam, Cambridge, MA), β-actin (1:5000; Santa Cruz Biotechnology, Santa Cruz, CA), and total H3 (1:1000; Cell Signaling Technology). IRDye 800-labeled secondary goat anti-mouse or goat anti-rabbit secondary antibodies (Li-Cor, Lincoln, NE) were added to bind to the primary antibody. The bound complex was detected using the Odyssey Infrared Imaging System (Li-Cor). The images were analyzed using the Odyssey Application Software, version 1.2 (Li-Cor) to obtain the integrated intensities.

### Co-immunoprecipitation of p53 and CRM1 proteins

Total protein extract from rat HCC JM1 cells was immunoprecipitated with p53 antibodies using a Dynabeads^®^ Protein G Immunoprecipitation kit (ThermoFisher Scientific). Briefly, p53 antibody (Abcam) was covalently coupled to the beads using BS3 (Thermo Scientific) as a cross-linker, according to the manufacturer's instructions. Total HCC JM1 cell extract was incubated with Dynabeads-Ab complexes for 10 min at room temperature to allow antigen binding. After elution of the bound p53-protein complexes by heating at 70°C, the samples were analyzed by Western blotting using anti-CRM1 and anti-p53 antibodies.

### Retrieval of data from online database

Gene expression data for the expression of *CRM1* gene and clinical and tumor pathological data were extracted as .txt files from The Cancer Genome Atlas database (TCGA; http://cancergenome.nih.gov).

### Statistical analyses

Results are presented as mean ± S.D. Comparisons between two groups were conducted with Student's *t*-test. Otherwise, data were analyzed by one-way analysis of variance (ANOVA), followed by Tukey's test for pair-wise comparisons. *P*-values < 0.05 were considered significant.

## Abbreviations

HCC: hepatocellular carcinoma
GST-P: glutathione-S-transferase placental form
CRM1: chromosome region maintenance 1
2-AAF: 2-acetylaminofluorene
DEN: N-nitrosodiethylamine
